# Mpox exposure on a congregate inpatient psychiatry unit: Description of the investigation and outcomes—New York City, 2022

**DOI:** 10.1017/ash.2023.315

**Published:** 2023-09-29

**Authors:** Waleed Malik, Justin Chan, Simon Dosovitz, Clyde Gilmore, Jeanne Cosico

## Abstract

**Background:** In May 2022, New York City (NYC) experienced a large outbreak of human mpox (clade IIb). Data on mpox transmission following exposure in healthcare facilities in nonendemic settings are limited. Because mpox was previously not seen in NYC, our healthcare staff may not always recognize a suspected case and therefore may neglect to implement timely infection prevention and control measures, leading to infectious exposures. The risk of transmission from unrecognized mpox may be higher in inpatient psychiatric units where direct physical contact is more common in the setting of common spaces for patients. In July 2022, a patient was admitted to NYC Health + Hospitals–Bellevue (Bellevue) psychiatry with signs and symptoms of mpox that were not recognized for 4 days, at which point the patient was tested for mpox and was isolated. We describe the investigation of staff and patients exposed during the 4 days prior to diagnosis and isolation of the index patient, and we report on the outcome mpox infection among those exposed. **Methods:** This study was a retrospective chart review of adult patients admitted to and staff working on an inpatient psychiatric unit where the patient with mpox was admitted to Bellevue, the largest municipal hospital in NYC. Each individual was classified regarding degree of exposure, based on criteria from the CDC, and was offered postexposure mpox vaccination where indicated. We describe the nature of contact with the patient for those with high-risk exposures. The outcome of interest was development of mpox infection during 21 days after last exposure. **Results:** In total, 29 patients and 84 staff members were identified to have been on the psychiatric unit prior to isolation of the index case of mpox. All exposed individuals were monitored for signs and symptoms of mpox for 21 days after last exposure. The exposed and unexposed patients were kept apart in the psychiatric unit. All patients who had contact were classified as having a low-to-intermediate risk exposure. Among 23 staff members exposed, 8 had high-risk exposures, 4 had intermediate-risk exposures, and 11 had low-risk exposures. Those with high-risk exposures were offered Jynneos as postexposure vaccination, but they declined. None of the exposed staff or patients developed mpox during the follow-up period. **Conclusions:** Mpox transmission was not observed despite several exposures in a congregate psychiatry unit. Given limited data, further studies are needed to better understand transmission risk in congregate healthcare settings.

**Disclosures:** None

